# Nationwide Epidemiology of Hospitalized Acute ACL Ruptures in Romania: A 7-Year Analysis (2017–2023)

**DOI:** 10.3390/medicina61091672

**Published:** 2025-09-15

**Authors:** Gloria Alexandra Tolan, Ionut Daniel Raducan, Bogdan Uivaraseanu, Delia Mirela Tit, Gabriela S. Bungau, Andrei-Flavius Radu, Cristian George Furau

**Affiliations:** 1 Multidisciplinary Doctoral School, “Vasile Goldis” Western University of Arad, 310414 Arad, Romania; gloria.tolan@yahoo.de; 2 Department of Pharmaceutical Sciences, “Vasile Goldis” Western University of Arad, 310414 Arad, Romania; 3 Department of Surgical Disciplines, Faculty of Medicine and Pharmacy, University of Oradea, 410073 Oradea, Romania; 4 Doctoral School of Biological and Biomedical Sciences, University of Oradea, 410087 Oradea, Romania; gbungau@uoradea.ro; 5 Department of Pharmacy, Faculty of Medicine and Pharmacy, University of Oradea, 410028 Oradea, Romania; 6 Department of Psycho-Neurosciences and Recovery, Faculty of Medicine and Pharmacy, University of Oradea, 410073 Oradea, Romania; andreiflavius.radu@uoradea.ro; 7 Department of Pathophysiology, Faculty of Medicine, “Vasile Goldis” Western University of Arad, 310414 Arad, Romania; furau.cristian@uvvg.ro

**Keywords:** anterior cruciate ligament rupture, knee injuries, epidemiology, incidence, sex differences, age distribution, Romania

## Abstract

*Background and Objectives:* Anterior cruciate ligament (ACL) rupture is one of the most frequent and debilitating knee injuries, especially among young, physically active individuals. While extensively studied in Western countries, large-scale epidemiological data from Eastern Europe remain scarce. This study offers the first nationwide assessment of hospitalization-based incidence of acute ACL rupture in Romania. *Materials and Methods:* We conducted a retrospective analysis of all hospital discharges coded as S83.53 (ACL rupture) between 2017 and 2023, using national public health datasets. Incidence rates were calculated per 100,000 inhabitants based on the 2021 national census. Data were analyzed by sex, age, year, and region. *Results:* A total of 4332 ACL-related discharges were recorded (3130 males and 1202 females), yielding an average incidence of 3.23 per 100,000 per year. Incidence in males was consistently higher (up to 5.63) than in females (up to 2.10). The peak incidence occurred in 2023, while the lowest was observed in 2020, likely due to COVID-19-related restrictions. Linear regression showed a significant upward trend over time (R^2^ = 0.966, *p* < 0.001). The highest age-specific incidence was found in males aged 25–29 years (116.3/100,000) and in females aged 15–19 years (35.4/100,000). Cases were rare above the age of 50. Geographically, incidence varied widely, with Bucharest, Timiș, and Bihor recorded the highest rates, while several other counties reported near-zero values. *Conclusions:* This study provides the first nationwide analysis of hospitalization-based ACL rupture incidence in Romania, revealing marked differences by age, sex, and region. While the findings reflect only acute cases requiring inpatient care, they underscore the need for more comprehensive injury surveillance, improved access to orthopedic services, and targeted prevention strategies tailored to high-risk populations.

## 1. Introduction

Anterior cruciate ligament (ACL) injuries are among the most frequent and clinically impactful knee conditions, particularly affecting young and physically active individuals [1,2]. Beyond the immediate pain and disability they cause, these injuries are often followed by long-term consequences such as joint instability, reduced athletic performance, and an increased risk of early-onset osteoarthritis [3]. In recent decades, the global incidence of ACL injuries has steadily risen [2], largely due to the growing popularity of high-impact and pivoting sports [2,4]. Epidemiological research consistently highlights clear differences between sexes, whereby women face a higher risk, influenced by hormonal, anatomical, and neuromuscular factors [5,6]. Age also plays a key role, with the highest incidence typically occurring in adolescents and young adults engaged in activities like soccer, handball, and skiing [7,8].

ACL rupture represents a pivotal event in the cascade of knee joint pathology. As the primary stabilizer for anterior tibial translation and rotational control, damage to the ACL compromises joint biomechanics, often resulting in recurrent instability, reduced proprioception, and poor functional performance [3]. Beyond its immediate mechanical consequences, ACL disruption initiates a pathological environment that predisposes the knee to secondary injuries such as meniscal tears, cartilage degeneration [9,10], and early-onset osteoarthritis [11]. These consequences are amplified by neuromuscular deficits that persist even after clinical recovery, accelerating degenerative change [12].

The broader burden of ACL injuries is not only clinical but also economic and societal. Systematic reviews have shown that long-term outcomes may include chronic pain, diminished quality of life, reduced participation in physical activity, and work limitations, translating into significant direct and indirect costs [13]. Advances in imaging techniques, particularly magnetic resonance imaging (MRI), have improved diagnostic accuracy and accelerated decision-making processes. Moreover, international clinical guidelines, such as those from the American Academy of Orthopaedic Surgeons (AAOS) and the European Society of Sports Traumatology, Knee Surgery and Arthroscopy (ESSKA), emphasize individualized treatment strategies tailored to the patient’s age, functional demands, and degree of joint instability [14,15].

While ACL injuries are among the most widely studied musculoskeletal conditions worldwide, there is a notable scarcity of large-scale epidemiological investigations in Eastern European countries, particularly Romania. The current understanding of incidence patterns, demographic risk profiles, and treatment pathways remains limited in this context. Moreover, national data are influenced by healthcare system factors such as unequal access to orthopedic care, regional disparities in surgical capacity, and variability in diagnostic or referral practices. These elements shape both the recognition and reporting of ACL injuries and highlight the need for robust population-based analyses to inform national clinical and policy strategies. In this context, the present study provides a nationwide overview of hospitalizations for ACL rupture in Romania over a seven-year period (2017–2023), based exclusively on inpatient discharge data coded as ICD-10 S83.53 [16]. The analysis focuses on incidence patterns stratified by sex, age, and region, aiming to identify major demographic and geographic trends. Although limited to acute inpatient cases, this study offers the first population-level assessment of hospitalization-based ACL rupture incidence in Romania. The observed regional and demographic disparities may reflect not only underlying injury patterns but also structural differences in access to orthopedic care and diagnostic infrastructure. These findings may serve as a foundation for improving national surveillance and guiding future prevention strategies.

## 2. Materials and Methods

### 2.1. Study Design and Data Sources

This study was designed as a retrospective descriptive analysis of hospitalizations for ACL ruptures in Romania over a 7-year period, from January 2017 to December 2023. The data were obtained from national hospitalization records (DRG system), which include all public and private hospitals that report discharge data to the national health authorities. The DRG system, although primarily designed for administrative and reimbursement purposes, represents the most comprehensive and standardized national source of hospitalization data currently available and can serve as a proxy for evaluating trends in injury-related admissions.

The analysis focused exclusively on discharges that included the diagnostic code S83.53, corresponding to a rupture of the anterior cruciate ligament, recorded as either a primary or secondary diagnosis. This ICD-10 code was selected because it most directly reflects acute ACL injuries requiring inpatient care and is widely used in hospital settings. Although ICD-10 code S83.53 may occasionally include acute-on-chronic presentations, it remains the most standardized and diagnostically specific classification for clinically significant ACL ruptures requiring hospitalization. All hospitalizations matching this diagnostic code were included in the analysis without applying additional inclusion or exclusion criteria. Due to the structure of the national DRG dataset, it was not possible to distinguish between primary and secondary diagnoses. As such, all discharges coded with S83.53 were included regardless of coding position, in order to capture the full hospitalization burden associated with ACL rupture.

Other potentially related codes, such as M23.2x (chronic ligament disorders), were excluded to preserve diagnostic precision and to avoid introducing heterogeneity related to coding variability and less specific clinical presentations. While this strategy limits the scope to acute hospital-managed injuries, it supports a clearer and more interpretable analysis of national trends based on consistent diagnostic labeling.

Hospitalization data were obtained from the National Institute of Public Health of Romania (no. 20606/5 December 2024), which compiles nationwide statistics from all public and private hospitals. The dataset consisted exclusively of anonymized, aggregate records and contained no personal identifiers. The study was conducted in compliance with the Declaration of Helsinki and relevant data protection regulations (GDPR, EU 2016/679).

### 2.2. Data Extraction and Processing

The initial dataset was received in aggregated form, structured by year, ICD10 diagnostic, county, hospital, age group, sex and total number of patients discharged. For this study, we selected only records that contained S83.53 and compiled all relevant data into a single Excel file for analysis. To improve the level of detail and allow for more specific epidemiological insights, a second file was created based on individual discharge-level records, with one row per hospitalization.

For each hospitalization record, the following variables were collected: year of hospitalization (2017–2023); patient sex (male/female); patient’s county hospitalization (all 41 counties plus Bucharest); age group (coded into 19 standard categories); and hospital name. The age was grouped according to the official DRG reporting structure in Romania, which includes 19 age categories, starting from “01—under 1 year,” followed by “02—1 to 4 years,” and continuing in 5-year intervals (e.g., 5–9, 10–14, …, 85+).

Each hospital was then manually classified as either public or private, based on the name of the institution.

### 2.3. Population Data and Incidence Calculation

To calculate incidence rates, population denominators were taken from the 2021 Romanian Population and Housing Census, published by the National Institute of Statistics [17]. These data included total population figures by county (Supplementary Table S1) and by age and sex (Supplementary Table S2) and were used as a constant reference for all incidence calculations, since annual projections were not consistently available for the entire study period. While this ensured consistency in denominator data, it may not fully capture year-to-year demographic changes, including migration and aging.

Incidence was calculated as the number of ACL rupture cases per 100,000 residents, using the standard formula recommended by the World Health Organization [18].

$$Incidence\;rate=\frac{no.\;of\;cases}{population}\times 100,000$$

Separate incidence values were determined for the total population, males, females, each of the 19 age categories, and each county (including Bucharest). All calculations and data organization procedures were conducted using Microsoft Excel.

### 2.4. Statistical Analysis

Descriptive statistical analyses were carried out in JASP (version 0.19.30) [19]. These included year-by-year case distributions, incidence comparisons by sex and age group and temporal trends from 2017 to 2023. For comparisons of categorical variables (e.g., sex distribution across age groups), the chi-square test was used. To evaluate whether incidence varied significantly over time or between sexes, we applied linear regression models, with statistical significance set at *p* < 0.05.

## 3. Results

### 3.1. General Overview of ACL Rupture Hospitalizations (2017–2023)

A total of 4332 hospitalizations were recorded nationwide in Romania for ACL rupture, as defined by ICD-10 code S83.53 [18]. Based on the total national population reported in the 2021 census (19,053,815 residents), this corresponds to a cumulative incidence of 22.7 per 100,000, and an average annual incidence of 3.23 per 100,000 inhabitants.

ACL ruptures were predominantly observed in male patients, with 3130 cases (72.3%), compared to 1202 cases (27.7%) in female patients, yielding a mean male-to-female ratio (M/F) of 2.6. This gender disparity was consistent across all years of observation (Table 1).

### 3.2. Temporal Trends and Sex Differences

Annual trends indicate a gradual increase in ACL-related hospitalizations from 2017 to 2019, peaking in 2019 with 681 cases. A sharp decline followed in 2020 (420 cases), likely due to COVID-19 restrictions on elective procedures. The trend recovered after 2021, with 2023 showing the highest recorded number of cases (727). The lowest incidence was recorded in 2020 (2.20/100,000), while the highest was in 2023 (3.82/100,000). Although males consistently represented most cases, the chi-square test of independence did not show a statistically significant variation in sex distribution over the years (χ^2^ = 3.117, df = 6, *p* = 0.794), indicating temporal stability in gender-related epidemiological patterns.

To assess the influence of time (Year) and sex (Group) on the incidence of ACL ruptures, a linear regression model was conducted, using incidence as the dependent variable (Table 2). The model showed significant contributions from both predictors (F = 42.611, *p* < 0.001).

**Figure 1:**
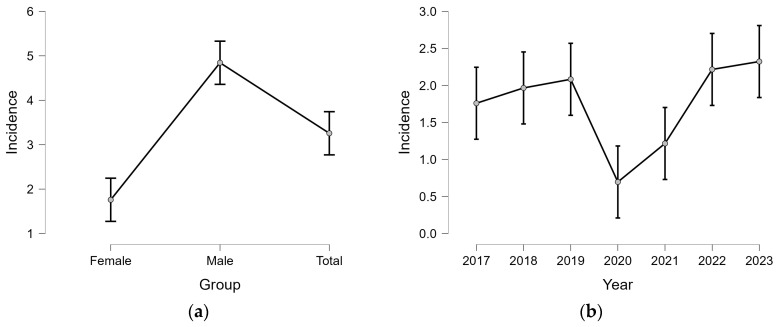
Marginal effects of sex and year on ACL rupture incidence: (**a**) Mean predicted incidence by sex and overall, based on linear regression with 95% confidence intervals; (**b**) Mean annual predicted incidence (per 100,000).

Linear regression modeling confirmed the significant effect of sex and year on ACL rupture incidence (R^2^ = 0.966, *p* < 0.001). Marginal effects plots (Figure 1a,b) illustrate the marked disparity between males and females, as well as the temporal dip in 2020 and subsequent rise in incidence.

### 3.3. Age and Sex-Specific Incidence Patterns (2017–2023)

The age-specific analysis of ACL rupture in the analyzed period reveals a significant concentration of cases among younger individuals. As shown in Table 3, the highest number of cases was recorded in the 20–24 and 25–29 age groups, which together accounted for 29.8% of all ACL ruptures. When extending this range to include individuals aged 20–34 years, the cumulative proportion rises to 46.1%.

Across nearly all age groups, incidence rates were consistently higher in males compared to females. The highest incidence was observed in males aged 25–29, reaching 116.27 per 100,000, followed closely by males in the 20–24 and 30–34 age groups, with rates of 101.42 and 90.71 per 100,000, respectively. In contrast, the highest incidence among females was observed in the 15–19 age group (35.41 per 100,000), followed by a gradual decline with increasing age. The overall male-to-female case ratio was 2.6:1, confirming a substantial sex-related disparity in ACL injury occurrence. This imbalance was particularly evident in the 25–34 age range, where the male-to-female ratio exceeded 4:1, peaking at 5.19:1 in the 25–29 group.

In age groups above 50 years, ACL rupture became increasingly rare, with all such groups combined accounting for less than 6% of total cases. In these groups, the sex ratio reversed slightly in some older categories, where the number of female cases began to exceed those in males.

A chi-square test of independence confirmed a statistically significant association between age group and sex distribution (χ^2^ = 290.041, df = 15, *p* < 0.001), highlighting that younger males represent the primary risk population for ACL rupture, while older age groups follow distinct patterns.

**Figure 2:**
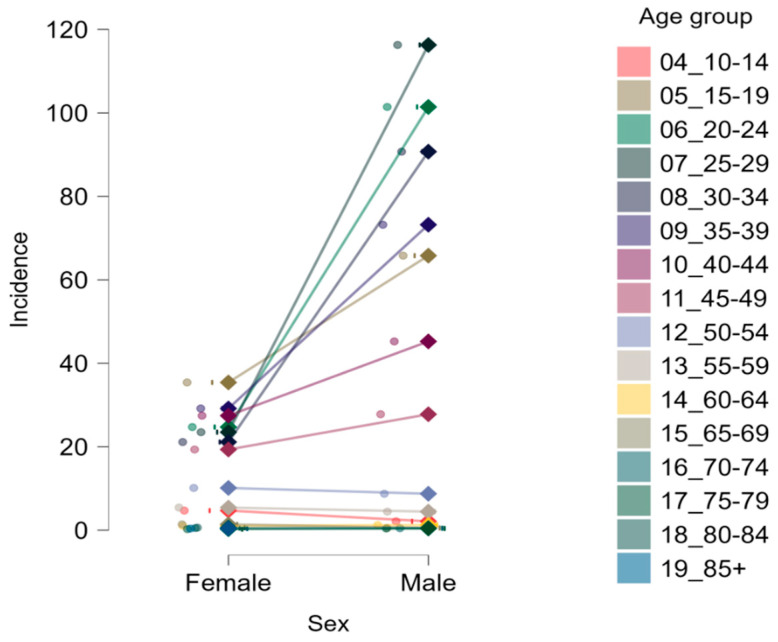
Sex-specific incidence of ACL rupture by age group. Values are expressed as cases per 100,000 population. Each line connects male and female incidence within the same age category, with 95% confidence intervals.

These trends are further illustrated in Figure 2, which depicts the age-specific incidence of ACL ruptures by sex. The steep increase from female to male incidence is most evident between 20 and 34 years. In older age groups, the sex gap narrows and inverts in some categories, suggesting a shift from traumatic to potentially degenerative etiologies.

**Figure 3:**
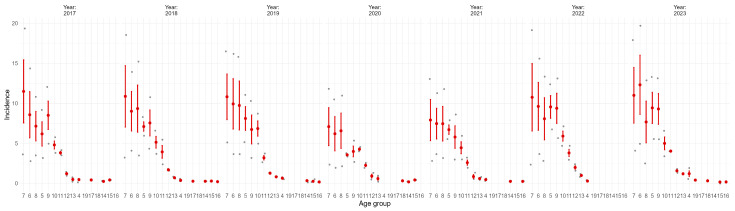
Annual incidence of anterior cruciate ligament ruptures, stratified by age group Each panel represents one calendar year. Values represent means with 95% confidence intervals. Age group codes correspond to the following intervals: 1 = 0 years, 2 = 1–4, 3 = 5–9, 4 = 10–14, 5 = 15–19, 6 = 20–24, 7 = 25–29, 8 = 30–34, 9 = 35–39, 10 = 40–44, 11 = 45–49, 12 = 50–54, 13 = 55–59, 14 = 60–64, 15 = 65–69, 16 = 70–74, 17 = 75–79, 18 = 80–84, 19 = 85+ years.

A detailed analysis of temporal trends revealed that, in each analyzed year the highest incidence rates of ACL rupture were consistently recorded in the 25–29 age group, followed closely by the 20–24 and 30–34 categories. This age-related distribution remained stable throughout the observation period, with only minor fluctuations across years (Figure 3).

**Figure 4:**
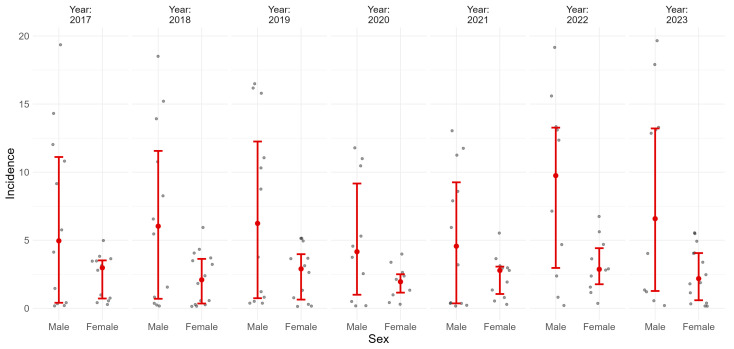
Annual incidence of ACL rupture by sex. Each panel represents one calendar year; values are shown with 95% confidence intervals.

To complement the age-specific temporal analysis, Figure 4 displays annual ACL rupture incidence by sex for the 2017–2023 period. In every year of the study, incidence was consistently higher among males, with a stable male-to-female gap across time. Although a drop in overall incidence occurred in 2020, the gender disparity persisted, reflecting a constant differential risk profile between sexes.

### 3.4. Geographic Distribution

**Figure 5:**
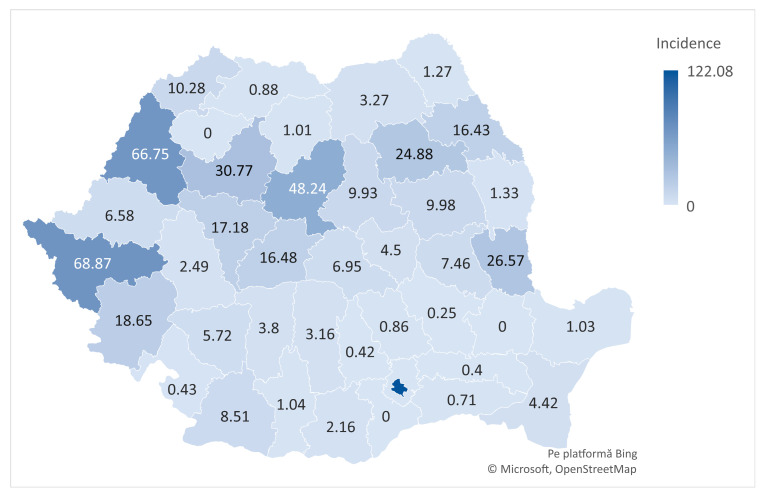
Geographic distribution of cumulative ACL rupture incidence in Romania, by county (2017–2023).

The spatial distribution of ACL rupture hospitalizations across Romanian counties during 2017–2023 demonstrated notable regional variability. The highest cumulative incidence was observed in Bucharest (122.08/100,000), followed by Timiș (68.87), Bihor (66.75), and Mureș (48.24), counties characterized by greater access to orthopedic care. Conversely, several counties, including Brăila, Giurgiu, and Călărași, reported either no cases or incidence rates below 1/100,000, suggesting potential underdiagnosis or limited access to diagnostic and surgical services (Figure 5).

**Figure 6:**
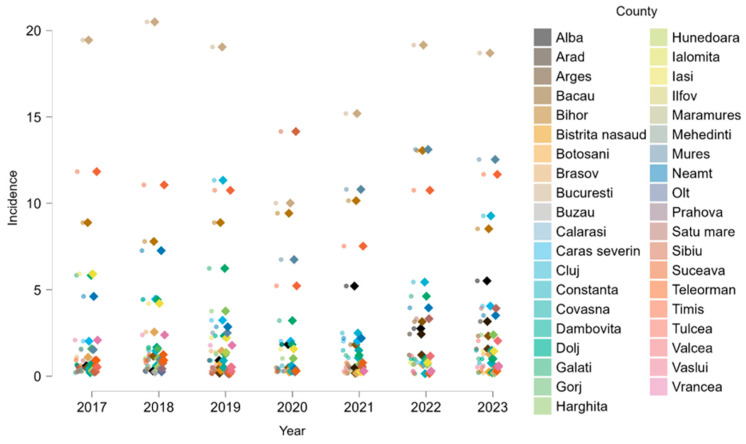
Annual incidence of ACL rupture by county.

To further explore temporal dynamics, county-level incidence rates were analyzed for each year of the study period. As shown in Figure 6, regional differences in ACL hospitalization persisted consistently over time. Counties with high cumulative incidence also showed sustained elevated rates annually, while many others remained consistently low. A general drop in incidence was observed in 2020, aligning with pandemic-related restrictions on elective procedures. This longitudinal perspective reinforces the stability of regional disparities in ACL injury burden across Romania.

**Figure 7:**
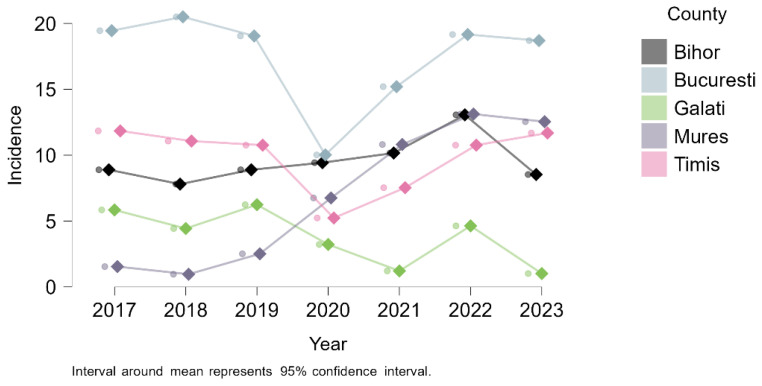
Annual incidence trends of ACL ruptures (per 100,000 inhabitants) in the top five counties (2017–2023).

Annual trends analysis for the five counties with the highest cumulative ACL rupture rates (Bucharest, Timiș, Bihor, Mureș, and Galați) revealed some differences. As shown in Figure 7, Bucharest consistently recorded the highest incidence, with a sharp decline in 2020, followed by a steady recovery. Timiș and Bihor followed a relatively stable trend with moderate fluctuations, while Galați showed more variable and generally lower incidence levels. Notably, Mureș did not experience a pandemic-related drop in 2020; instead, it showed a continuous increase throughout the study period, reaching its highest incidence in 2022. These distinct trajectories highlight local differences in healthcare access, diagnostic practices, and the impact of external factors such as the pandemic.

These counties together account for 76.54% of all cases nationwide and include both major urban centers and regional orthopedic hubs. Analysis of the healthcare sector distribution revealed significant variation in the use of public versus private hospitals for ACL rupture hospitalizations. In Bihor County, nearly 79% of all recorded cases were treated in private facilities. This contrasts sharply with Bucharest, where the vast majority (89%) of cases occurred in public hospitals, highlighting the dominant role of the public healthcare system in the capital. In Galați County, all ACL cases were managed exclusively in public institutions (Table 4).

## 4. Discussion

This nationwide analysis provides an essential first step in characterizing the epidemiology of anterior cruciate ligament (ACL) rupture hospitalizations in Romania. By focusing on a single, clearly defined ICD-10 code (S83.53), the study offers insight into the demographic and regional distribution of cases requiring inpatient care, a subset likely to reflect more severe injuries and those considered for surgical treatment. The patterns identified, particularly the age and sex distributions, as well as regional clustering, are consistent with findings reported in international literature, but also reflect the specific structure of the Romanian healthcare system. To the best of our knowledge, this is the first nationwide study to describe the epidemiology of ACL rupture in Romania, using discharge diagnosis data from continuous inpatient care in both public and private hospitals.

Between 2017 and 2019, acute ACL rupture hospitalizations in Romania increased gradually, consistent with international evidence linking such trends to greater participation in high-risk sports, improved access to orthopedic care, and advances in diagnostic capabilities [20,21,22]. A sharp decline followed in 2020, with incidence falling by approximately 40% compared to 2019, coinciding with the COVID-19 pandemic and its restrictions on elective procedures and organized sports. Similar reductions have been documented globally, attributed to the suspension of competitive activities and the postponement of non-emergency surgeries. From 2021 onward, incidence rebounded, and by 2023 it exceeded pre-pandemic levels, suggesting a possible “post-pandemic surge” driven by detraining effects and a rapid return to sport. However, this increase may also reflect delayed presentation of ACL injuries sustained during the pandemic period, when elective procedures were widely suspended and many patients postponed medical evaluation due to healthcare restrictions [23,24].

Over the entire study period, the mean annual incidence of ACL rupture hospitalization was 3.23 per 100,000 inhabitants, markedly lower than values reported in many Western European and North American studies [25,26,27,28]. In the United States, for instance, the ACL is the most injured knee ligament, with an estimated annual incidence of 1 in 3500 people and about 400,000 reconstructions performed each year [28,29]. This difference must be interpreted in the context of our methodology. The present analysis is based on hospital discharge records for continuous inpatient care coded as S83.53 and therefore captures only cases requiring hospitalization. Injuries managed entirely in outpatient settings, unreported cases, or those occurring in regions with limited access to MRI and surgical intervention are not reflected in these figures. Consequently, the true burden of ACL rupture in Romania is likely higher, particularly in counties with limited orthopedic capacity or among patients who seek care in private or non-contracted facilities. Direct comparison with Western European or North American studies should be interpreted with caution, as those often include broader case definitions based on outpatient diagnoses, MRI confirmation, and sports-related clinical encounters. Our hospitalization-based approach prioritizes diagnostic clarity and national coverage but may exclude milder or conservatively managed cases. Additionally, limitations in ICD coding granularity prevent differentiation between primary acute ruptures and acute-on-chronic presentations, introducing some heterogeneity. Nevertheless, all included cases represent clinically significant ACL pathology that warranted inpatient orthopedic care.

A pronounced male predominance was observed, with men accounting for 72.3% of all cases and a stable male-to-female ratio of approximately 2.6:1 across the study period. This imbalance was most evident in the 20–34 age range, where over 80% of cases were male, likely reflecting higher engagement in pivot-intensive and contact sports such as football and basketball [30]. Nevertheless, females are biomechanically more susceptible to ACL injury during equivalent athletic activity, with reported risks two to eight times higher than in men in sports like basketball or soccer [31,32]. In our dataset, females predominated in the 10–14 age group and in patients over 50 years, suggesting different injury mechanisms, possibly related to growth and hormonal changes in adolescents, and to degenerative and hormonal changes [33], domestic trauma, or delayed presentation in older adults.

Geographic analysis revealed marked regional disparities. ACL-rupture hospitalizations were concentrated in Bucharest and just a few counties (Timiș, Bihor, Mureș, and Galați) together accounting for more than three-quarters of all cases nationwide. These counties include major referral centers in cities such as Bucharest, Timișoara, Oradea, Târgu Mureș, and Galați, where advanced imaging, arthroscopic surgery, and specialized sports medicine services are available. This concentration is consistent with patterns reported in other European settings, where ACL treatment is often centralized in high-volume centers with specialized expertise [34]. Differences in how care is delivered also play a role: in Bihor, almost 80% of cases were treated in private hospitals, while in Bucharest and Galați the public sector dominated (76.2% and 100%, respectively), reflecting variation in healthcare infrastructure and patient pathways [35]. This geographic concentration likely reflects referral patterns and diagnostic availability more than actual differences in injury prevalence. Underdiagnosis or delayed presentation in rural areas may contribute to observed regional gaps. Healthcare disparities between urban and rural regions in Romania are well documented, with rural populations facing limited access to specialist care, advanced imaging (such as MRI), and surgical infrastructure [36]. These systemic barriers likely influence both diagnostic rates and thresholds for hospitalization in ACL cases, potentially distorting the true geographic distribution of injury burden.

The long-term consequences of ACL injury are substantial, regardless of whether reconstruction is performed. Meta-analyses show that radiographic knee osteoarthritis (OA) develops in 20–50% of ACL-injured knees within 10–15 years, with risk increasing over time [37,38,39] and serious impact on the patients ‘quality of life [40,41]. In one large study, the incidence of OA 14 years after ACL reconstruction was 57% in the injured knee compared with 18% in the contralateral knee [39]. Knees that did not undergo reconstruction had an even higher relative risk of OA than those treated surgically [38,42,43]. Beyond structural degeneration, many individuals report persistent knee pain, reduced recreational capacity, and impaired quality of life (QOL) more than five years post-injury [38]. Recurrent ACL injuries, which are not uncommon, are associated with poorer long-term outcomes [44]. Collectively, these factors contribute to a significant and sustained burden, both in terms of individual QOL and public health, highlighting the need for targeted prevention, early intervention, and personalized rehabilitation strategies to optimize long-term outcomes [45,46].

Prevention programs offer a proven strategy to reduce incidence, particularly among female athletes. Neuromuscular training interventions, such as FIFA 11+ and PEP, have demonstrated 50–67% reductions in ACL injury risk [47,48]. Given the centralized nature of orthopedic care, and rising participation of Romanian youth in organized sports, the national implementation and scaling of such evidence-based prevention initiatives, coupled with awareness campaigns and regional capacity building in arthroscopic knee surgery, should be a priority. Without these measures, Romania may face a growing burden of avoidable injury, escalating costs, and premature disability in otherwise healthy young adults.

Despite its inherent limitations, this study offers the most comprehensive overview to date of ACL rupture epidemiology in Romania. Its primary strengths lie in its nationwide scope, seven-year observation period, and use of official hospitalization data reported by all public and private healthcare institutions. The combination of aggregate and individual-level records enabled detailed stratification by age, sex, and geographic region, capturing both temporal and spatial patterns with a high degree of resolution.

While the study offers a broad national overview, several important limitations must be considered. The analysis is based exclusively on hospital discharge data, which tends to reflect more severe cases, typically those requiring surgical intervention. As a result, ACL injuries managed conservatively in outpatient settings, undiagnosed cases, or those occurring in underserved regions may be underrepresented. The study focused solely on hospitalizations coded with ICD-10 code S83.53, corresponding to acute ACL rupture. Other potentially related conditions, such as chronic ligament disorders (e.g., M23.2X), were excluded to preserve diagnostic clarity and reduce variability in clinical interpretation and coding practices. The dataset was derived from Romania’s national DRG system, which, despite being standardized and nationwide in scope, was primarily developed for administrative and reimbursement purposes. This may lead to inconsistencies in coding practices across hospitals, and thus the data should be interpreted as a structured proxy rather than a fully comprehensive epidemiological record. Moreover, due to the structure of the dataset, it was not possible to differentiate between cases where ACL rupture was recorded as a primary diagnosis versus a secondary one. This means that hospitalizations in which ACL injury was a secondary finding, such as in cases of polytrauma or complex knee injuries, could not be analyzed separately, introducing potential heterogeneity in the clinical severity and context of the included cases. Similarly, we were unable to validate diagnostic codes against imaging or surgical records, which introduces a degree of uncertainty related to coding accuracy. ICD-10 code S83.53 may also encompass both acute traumatic ruptures and acute-on-chronic presentations, which represent different clinical entities. However, the available data did not allow us to distinguish between these subtypes, nor to confirm diagnoses at the individual level. Surgical procedures are recorded in a separate national registry that could not be linked to the diagnostic dataset. Moreover, the absence of treatment data limits the interpretation of geographic disparities, as it remains unclear whether these reflect differences in injury incidence, surgical capacity, or clinical decision-making. Finally, population estimates were drawn from the 2021 national census and applied uniformly across the entire 2017–2023 period. While this approach ensured consistency, it may not fully capture demographic shifts over time, such as emigration or population aging. Age data were grouped in standard 5-year intervals, limiting more granular analysis, particularly in high-risk groups like adolescents and young adults. Additionally, incidence rates were calculated based on the county of hospitalization rather than the patient’s residence, which could overestimate incidence in urban referral centers while underestimating it in more rural or remote areas.

Despite these limitations, the dataset revealed stable patterns across time and regions, providing a meaningful baseline for understanding ACL rupture hospitalizations in Romania and a starting point for more integrated national surveillance and prevention efforts.

## 5. Conclusions

This study provides a comprehensive national overview of anterior cruciate ligament (ACL) rupture hospitalizations in Romania, based on seven years of standardized data reported by all public and private hospitals. By focusing on inpatient cases coded as S83.53, the analysis captures a well-defined subset of acute ACL injuries that typically require surgical care. Although the study does not include outpatient or chronic cases, it reveals consistent patterns by age, sex, and region. The majority of hospitalizations occurred in young, active males aged 20–34, while regional differences point to significant inequalities in access to orthopedic services, particularly advanced imaging and surgical intervention.

The findings offer a valuable starting point for future research and health planning. They can support the development of national injury surveillance systems, guide more equitable distribution of orthopedic resources, and inform prevention strategies for high-risk groups. Looking ahead, integrating procedural data, outpatient care records, and long-term outcomes will be essential for capturing the full burden of ACL injuries in Romania.

## Figures and Tables

**Table 1:** Annual number of ACL rupture hospitalizations (ICD-10 S83.53) by sex and year.

Year	Total Cases	Male	Female	M/F Ratio	Incidence (/100,000)		Total Incidence (/100,000)
					Male	Female	
2017	620	451	169	2.67	4.88	1.72	3.26
2018	660	480	180	2.67	5.19	1.83	3.46
2019	681	500	181	2.76	5.41	1.85	3.57
2020	420	292	128	2.28	3.16	1.31	2.20
2021	518	368	150	2.45	3.98	1.53	2.72
2022	706	518	188	2.76	5.60	1.92	3.71
2023	727	521	206	2.53	5.63	2.10	3.82
Total	4332	3130	1202	2.60	–	–	3.23

**Table 2:** Results of linear regression model evaluating the effects of year and sex group on annual incidence rates of ACL rupture (per 100,000 inhabitants).

Predictor	Unstandardized Coefficient	Standard Error	t Value	*p* Value
Intercept	1.76	0.223	7.886	<0.001
Group (Male)	3.084	0.182	16.93	
Group (Total)	1.497	0.182	8.218	
**Year**				
2018	0.207	0.278	0.743	0.472
2019	0.323	0.278	1.162	0.268
2020	−1.063	0.278	−3.821	0.002
2021	−0.543	0.278	−1.952	0.075
2022	0.457	0.278	1.641	0.127
2023	0.563	0.278	2.024	0.066

**Table 3:** Age and sex-specific distribution and incidence of ACL ruptures in Romania (2017–2023).

Age Group (Years)	Cases		Incidence		Total Cases	M/F Ratio	Total Incidence
	Female	Male	Female	Male			
04_10–14	25	12	4.71	2.13	37	0.48	3.38
05_15–19	179	351	35.41	65.79	530	1.96	51.01
06_20–24	115	495	24.72	101.42	610	4.3	63.99
07_25–29	110	571	23.49	116.27	681	5.19	70.98
08_30–34	128	579	21.13	90.71	707	4.52	56.83
09_35–39	176	469	29.17	73.2	645	2.66	51.84
10_40–44	194	337	27.45	45.24	531	1.74	36.58
11_45–49	139	208	19.34	27.79	347	1.5	23.65
12_50–54	84	73	10.13	8.74	157	0.87	9.43
13_55–59	28	22	5.42	4.46	50	0.79	4.95
14_60–64	7	7	1.06	1.25	14	1	1.15
15_65–69	10	3	1.41	0.54	13	0.3	1.03
16_70–74	3	2	0.52	0.48	5	0.67	0.5
17_75–79	1	1	0.26	0.42	2	1	0.32
18_80–84	2	0	0.62	0	2	0	-
19_85+	1	0	0.4	0	1	0	-

**Table 4:** Distribution of ACL rupture hospitalizations by healthcare sector in top counties (2017–2023).

County	Total Cases	Hospitals			
		Public		Private	
	n	n	%	n	%
Bihor	368	74	20.11	294	79.89
Bucharest	2096	1598	76.24	498	23.76
Galati	132	132	100.00	0	0.00
Mures	250	245	98.00	5	2.00
Timiș	448	410	91.52	38	8.48

## Data Availability

Data are contained in the manuscript. More information should be requested from the first author.

## Supplementary Materials

The following supporting information can be downloaded at: https://www.mdpi.com/article/10.3390/medicina61091672/s1, Table S1. County-level population data from the 2021 Romanian Population and Housing Census, used as denominators in anterior cruciate ligament (ACL) rupture incidence calculations; Table S2. Population data by age group and sex from the 2021 Romanian Population and Housing Census, used as denominators in anterior cruciate ligament (ACL) rupture incidence calculations.

## Abbreviations

The following abbreviations are used in this manuscript:

ACL	Anterior cruciate ligament
MRI	Magnetic resonance imaging
ICD-10	International Classification of Diseases, 10th Revision
M/F	Male-to-Female Ratio
ESSKA	European Society of Sports Traumatology, Knee Surgery and Arthroscopy
AAOS	American Academy of Orthopaedic Surgeons
COVID-19	Coronavirus Disease 2019
